# Attitudes of Healthcare Workers about Prevention and Control of Nosocomial Multidrug-Resistant Tuberculosis Infection in Two Top-Ranked Tuberculosis Specialized Public Hospitals of Ethiopia

**DOI:** 10.1155/2022/5266347

**Published:** 2022-12-14

**Authors:** Taye Kebede, Million Molla Sisay

**Affiliations:** ^1^Department of Biomedical Sciences and Immunology, Natural Sciences College, Madda Walabu University, P.O. Box 247, Bale-Robe, Ethiopia; ^2^Aklilu Lemma Institute of Pathobiology, Addis Ababa University, P.O. Box 1176, Addis Ababa, Ethiopia; ^3^Department of Research and Evidence Generation Directorate, Saint Peter's TB Specialized Hospital, Addis Ababa Administration City Health Bureau, Addis Ababa, Ethiopia

## Abstract

**Background:**

Tuberculosis (TB) exists as a human curse since antiquity. Around 9.5 million cases and 1.5 million deaths were reported due to TB in 2021. Ethiopia is one of the high-burdenmultidrug-resistant (MDR) TB countries. MDR-TB is acquired either by poor adherence to treatment or by primary infection with a drug-resistant strain, which has a high transmission rate from patients to healthcare workers (HCWs). Hospital outbreaks of MDR-TB are common in Africa. Hence, this study aimed to score the attitude of HCWs working in the two nationally top-rankedTB-specialized hospitals in Ethiopia, Saint Peter's and ALERT TB-specialized public hospitals about the infection prevention and control (IPC) of nosocomial MDR-TB.

**Methods:**

A cross-sectional study was conducted from December 1, 2020, to March 31, 2021. A simple random sampling method was applied to select 384 HCWs. The data collection tool was a self-administered interview structured questionnaire. The data were analyzed using SPSS software. Descriptive statistics were applied to score attitude. Bivariate and multivariable logistic regression models were performed to identify the independent determinants of attitude. The odds ratio was used to test the degree of association between variables at a 95% confidence interval (CI). The level of statistical significance was fixed at *p* value < 0.05.

**Results:**

Among the respondents, 87% of the HCWs held favourable attitudes about the nosocomial MDR-TB-IPC. The favourable attitude score had a significant association with the monthly salary earned between 7001 and 9000 ETB (Ethiopian Birr) (AOR = 3.34, 95% CI: 1.11, 10.05) and the previous training obtained on TB/MDR-TB (AOR = 2.96, 95% CI: 1.32, 6.62).

**Conclusions:**

Almost one in seven HCWs has an unfavourable attitude. Prior training received and earning monthly income above 7000 ETB are independent determinants of a favourable attitude score. Refreshment training and a reasonable increment in monthly income should be strengthened in TB-specialized hospitals in Ethiopia.

## 1. Introduction

Populations in resource-limited settings account for nearly 95% of *Mycobacterium tuberculosis* infections [1], as healthcare settings in resource-rich countries have enjoyed tremendous success in the prevention of healthcare-associated TB transmission [2]. Nosocomial infections are healthcare-associated infections or infections occurring in healthcare facilities, including hospitals, representing the most frequent adverse event associated with patient care [3]. The TB epidemic is complicated by multidrug-resistant tuberculosis (MDR-TB), which is a man-made disease that emerged as a result of inadequate TB treatment [4].

Currently, drug-resistant TB continues to be a global health threat and is elaborated by higher morbidity and mortality, sequelae, and higher cost and complexity. The World Health Organization (WHO) classifies drug-resistant TB into 5 categories: isoniazid-resistant TB; rifampicin-resistant (RR)-TB; MDR-TB (TB-resistant to isoniazid and rifampicin); preextensively drug-resistant TB (pre-XDR-TB), which is MDR-TB with resistance to a fluoroquinolone; and finally XDR-TB that is TB-resistant to rifampicin, plus any fluoroquinolone, plus at least one further priority A drug (bedaquiline or linezolid). Of 500,000 estimated new cases of RR-TB in 2020, only 157, 903 cases are notified. More havoc about it is that, only about a third of cases are detected and treated annually [5].

The need to initiate all TB patients on appropriate therapy and to ensure relapse-free survival is a key foundation of efforts to combat TB today. Given this reliance on therapeutic strategies, drug resistance poses a critical issue. Such forms of TB are both difficult to diagnose and treat. Although MDR-TB accounts for less than 6% of the global TB burden, patient management to treat it still accounts for over a quarter of programmatic spending on TB worldwide [6]. Although there has been a decrease in TB incidence and mortality in recent years, we are still far from the global TB targets proposed by the WHO in its “End TB” strategy launched in 2015 that aims to reduce the absolute number of TB deaths by 95% and the absolute number of new cases by 90% by 2035 [7].

Employee occupational health programs promote a safe and healthy workplace. This is accomplished by minimizing any exposures, promptly detecting and treating exposures, and using information gained from health institutions' incidents and accidents to enhance safety precautions. Theoretically, a baseline medical checkup and provision for regular follow-up are considered for all staff before they commence work in the TB clinics and laboratories. The healthcare providers providing occupational health services are expected to be knowledgeable about the nature of potential health risks in TB clinic facilities and laboratories, as well as have access to experts for consultation. Medical services are readily available to allow timely and appropriate evaluation and treatment [8]. In addition, laboratories in TB/MDR-TB-treating health facilities need to have sufficient space to ensure the quality, safety, and efficiency of the services provided to clients whose samples are tested and to ensure the safety of HCWs, patients, and visitors [9].

The role of healthcare institutions in the propagation of the TB and MDR-TB outbreaks provides concrete evidence of how the concentration of immunosuppressed patients on open wards facilitates the spread of TB, MDR-TB, and XDR-TB [10]. A large burden of potentially infectious TB among inpatients and undiagnosed MDR-TB among patients dying at hospitals represent the nosocomial transmission of TB in general and MDR-TB in particular [11].

According to the Sustainable Development Goals (SDGs) Report 2022, in 2020, an estimated 10 million people worldwide fell ill with TB. That year, the notification rate of new and relapse cases in TB incidence fell to 59%, down from 72% in 2019. Disruptions associated with the pandemic globally caused a noticeable rise in the number of TB deaths, from 1.2 million in 2019 to 1.3 million in 2020 (excluding TB deaths in people with HIV/AIDS). Between 2018 and 2020, TB treatment reached 20 million people, only half of the global target. Due to the COVID-19 disease pandemic, TB incidence and mortality are expected to worsen, especially in 2021 and 2022 [12].

Besides deteriorating health, antimicrobial resistance has impacts on environmental, social, and economic targets in the SDG framework. Hence, it needs greater international collaboration and accountability distribution and a broader engagement of countries and United Nations agencies to foster global intersectoral action on antimicrobial resistance. Drug-resistant pathogens can reverse the positive trend (before COVID-19 emergence) of falling global mortality rates from infectious diseases, which have decreased from 23% to 17% of total deaths over 15 years [13]. Especially, drug-resistant nosocomial infections not only represent patients' infections at facilities but also occupational infections among staff members. Particularly in Africa, healthcare-associated infections are more frequent and cause significant damage [14].

Healthcare workers have an increased risk of infection due to occupational exposure to MDR *Mycobacterium tuberculosis* (MTB) strains [15]. The risk of developing tuberculosis is higher by 10–20 folds over the general population, irrespective of the incidence rate in a given country [16]. Because of an occupational hazard, healthcare workers are inevitably exposed to TB due to frequent interaction with patients who have undiagnosed and potentially contagious agent-carrier individuals, specifically if there is no strict implementation of infection prevention and control (IPC) practices and protocol breaches [17].

In 2016, the WHO reported an estimated risk of two-to-three-fold greater transmission of nosocomial TB infections than the general population in low- and middle-income countries (LMICs) among HCWs [18]. Patients who had failed previous TB treatment, relapsed after treatment, contacted known MDR-TB patients, the patient who defaulted during previous treatment, and HIV patients are the potential suspect of contracting MDR-TB. The transmission risk of MTB from patients to healthcare workers (HCWs) is ignored in many LMICs, as most of these countries lack the necessary resources to prevent nosocomial transmission of MDR-TB [19]. Both mismanagement of TB treatment and person-to-person transmission are the primary reasons for the continued emergence and spread of MDR-TB [20].

Most nosocomial infections are prevented by adopting inexpensive infection prevention and control measures. In many healthcare facilities, there is a need for functional surveillance programs, a continuous supply of antiseptics, a safe water supply, personal protective equipment, essential antibiotics to treat infections, an appropriate number of health care personnel trained in infection control, appropriate health care infrastructure, and political commitment [21]. More specifically, surveillance of high-risk HCWs and appropriate infrastructure modifications are important to prevent interpersonal MDR-TB transmission in healthcare facilities, as a body mass index of less than 19 kg/m^2^ and working in medical wards and microbiology laboratories raise the risk of acquiring MDR-TB infections among HCWs in India [22].

In Ethiopia, there are two TB-specialized hospitals, namely, Saint Peter's and ALERT TB-specialized hospitals, known for admitting and treating MDR-TB patients from all corners of the country, which are known for the country's MDR-TB Treatment Initiating Centers (TICs). Like the other LMICs, infection control procedures for suspected and active MDR-TB cases in Ethiopia are linked to the poor infrastructure set-up (poor ventilation, lack of individual rooms, inability to pinpoint patients with MDR-TB), the shortage of personal protective equipment (N95 respirator), and lower laboratory diagnostic capacity for TB [19]. Patients with suspected or active TB are admitted to the common ward without any special attention to TB status until their diagnostic procedures are finalized, leading to the mixing up of patients and the obvious HCWs in different wards [23].

A decade ago, most of the HCWs in the general purpose health institutions of the capital city of Ethiopia felt that they were at high risk for occupational acquisition of TB (71%). But, only 12% of those HCWs regularly used to wear masks whenever engaged in caring activities for TB patients [24]. The Federal Ministry of the health of The Democratic Republic of Ethiopia developed the national TB/HIV implementation guideline in 2005 to guide healthcare providers to deliver effective TB and HIV/AIDS collaborative activities [25]. Although the Ethiopian HCWs are very important stakeholders in the MDR-TB care delivery, their attitude towards nosocomial MDR-TB-IPC in health institutions daily affects the livelihood of the HCWs and their guardians. In this regard, there are limited published study findings in Ethiopia, particularly the TB-specialized public hospitals serving the larger populations of the country through referral destinations.

Protecting the scarce health professionals from this highly contagious MDR-TB disease and sparing the longer and tiresome treatment duration have paramount importance in TB national treatment hospitals. Complementing the research gap in these areas is hoped to support the policy formulators for decision-making. So, it is instrumental to assess the existing IPC of HCWs about MDR-TB in TB treatment centres and its independent predictors. Therefore, this study aimed to assess the attitude of healthcare providers working at Saint Peter's and ALERT TB-specialized public hospitals of Ethiopia about the prevention and control of nosocomial MDR-TB infection and its associated determinant factors.

## 2. Materials and Methods

### 2.1. Study Setting

The study was conducted in Saint Peter's and ALERT TB Specialized Public Hospitals of Ethiopia. Both hospitals serve as the main destination for referred MDR-TB suspected and confirmed cases of TB patients from all corners of the country, and both are found in the capital city of Ethiopia, Addis Ababa. St. Peter's TB-specialized hospital was established in 1953 as the first national TB hospital and the first MDR-TB treatment centre since 2017. The hospital has a total employee of 908 (619 technical staff/HCWs and 289 supporting staff). On the other hand, the All-African Leprosy and Tuberculosis Rehabilitation and Training Center (ALERT) was established in 1934 to serve as a specialized clinical research and training centre for Ethiopia, Africa, and beyond for *Mycobacterium* bacterial infections (TB and leprosy) and other related infectious diseases. The ALERT hospital has a total employee population of 1300 (560 technical staff/HCWs and 740 supporting staff). Both hospitals have a MDR-TB Treatment Initiating Center (TIC) and a Treatment Follow-up Center (TFC) and are known to be the only centres to admit and treat MDR-TB patients in Ethiopia.

### 2.2. Study Design and Period

A cross-sectional study was conducted from December 1, 2020, to March 31, 2021, at Saint Peter's and ALERT TB-specialized public hospitals in Ethiopia.

### 2.3. Source and Study Populations

The source population was all healthcare workers working at Saint Peter's and ALERT TB Specialized public hospitals. The study population was the selected healthcare workers who had been working in the year preceding the study period at the TB/MDR-TB departments, both inpatient department (IPD) and outpatient department (OPD), such as triage officers, radiography personnel, pharmacists, nurses, medical laboratory technologists (MLT), midwives, and doctors of the hospitals, supporting staff such as janitors, runners, food servers, oxygen maintenance staffs, OPD finance staff, and DOTS supporters who have direct contact with the patients and volunteer to fill and return the questionnaires.

### 2.4. Inclusion and Exclusion Criteria

The inclusion criteria were HCWs of both hospitals whose age is at least 18 years and duties are linked with the TB patients service delivery and/or TB wards including medical doctors (59 out of 181), health officers (15 out of 48), nurses (164 out of 502), radiology personnel (14 out of 43), pharmacy experts (28 out of 86), MLT (35 out of 107), midwives (15 out of 46), and 25 (out of 77) some other workers (fewer in number among the staff profile) including health informatics educators, triage officers, and DOTS supporters. Additionally, a total of 29 (out of 89) janitors, runners, food servants, oxygen maintenance staffs, and OPD finance staff personnel were included. The exclusion criteria were staff members working in the hospital whose jobs are far away from the wards and OPD employees such as administrative staff and other office workers.

### 2.5. Sample Size Determinations

We could not find and access any published study conducted on infection prevention and control in TB Specialized public hospitals within Ethiopia and elsewhere to use it as a benchmark sample size calculation. As a result, we employed the epidemiological amenable sample size estimation formula by using a single population proportion formula for large finite samples, as follows:

(1) $$\begin{array}{c} Sample\;size\left(n\right)={\frac{1}{d^{2}}\left(Z_{\propto /2}\right)}^{2}P\left(1-P\right)\text{,}\\ n={\frac{1}{{\left(0.05\right)}^{2}}\left(1.96\right)}^{2}0.486\left(0.514\right)=383.86\text{,} \end{array}$$

where (***ρ*)** represents the proportion of attitude score of healthcare workers about nosocomial MDR-TB-IPC, (***ρ***) = 0.486, (**1-*ρ***) = 0.514, (**d)** = marginal of error = 0.05, (**Z**_***α*/2**_) = 95% level of confidence with (**Z**) = 1.96, and (**ո)** represents the calculated number of HCWs included in the study. Using the indicated formula, the calculated sample size was 383.86 (384 HCWs).

### 2.6. Sampling Method

A stratified sampling method was employed by giving attention to the number of staff profiles engaged in TB/MDR-TB associated management and treatment in both TB-specialized public hospitals. The Saint Peter's Hospital staff is fully dedicated to TB-related duties while that of ALERT hospital is for both Mycobacterial infection (TB and leprosy) duties. Hence, we recruited two-thirds of the study population from Saint Peter's TB-specialized hospital while the remaining HCWs from ALERT hospitals by using random sampling methods at the individual HCW level from both hospitals. The nonresponse rate was brought to zero, by tolerating a reasonable extension of the questionnaire filled out to be returned.

### 2.7. Study Tools and Variables

Data were collected using a self-administered questionnaire. It was developed through an in-depth literature review of other related previous research works. The attitude questions were adapted from MDR-TB-IPC guidelines and customized for HCWs working at TB-specialized hospital. The questionnaire was prepared originally in English and translated into the Amharic language in consultation with a language expert from the Language Studies College of Addis Ababa University. Later, during the data entry period, the Amharic language version was back-transcribed into the English language version. Also, all four data collectors were all health professionals; health officers, nurses, MLT, and health education specialists.

The dependent variable measured in this study was the attitude of HCWs about nosocomial MDR-TB-IPC in accordance with the Ethiopian standard practice procedures for TB infection control [25] and included TB infection control questions around healthcare facilities and HCWs [26]. Our study tool/instrument, the questionnaire for the HCWs, mimics that of the protocol reported from China in 2018 [26], was designed to elicit information on demographics, duration of employment at TB/MDR-TB facilities, professional category, length of service in current position, prior history of training to conduct TB/MDR-TB infection control, smoking history, previous and current history of TB/MDR-TB infection, and details of laboratory results and treatment whenever the history of infection with TB/MDR-TB.

The attitude is assessed with 9 questions, based on the Likert scale. Scoring was conducted by classifying the responses into two groups. If the right response is agreed or strongly agree, then a score of 1 is given; otherwise, zero. If the right response is disagreed or strongly disagree, then a score of 1 is given; otherwise, zero. The question covered areas of their protection, reducing the risk of transmission, patients' education, community awareness, their fear of contracting the disease, social and cultural factors, traditional medication, treatment, and MDR-TB curability. It was categorized as favourable when the scoring is equal to or above the mean, otherwise an unfavourable attitude. Favourable attitude indicating that the HCWs are afraid of contracting the disease while treating patients, assert that patients did not cause the problems, and support the patients without discrimination/blame. On another hand, the independent variables evaluated were **s**ociodemographic variables (age, sex, and marital status), socioeconomic variables (monthly income, type of profession, and educational status), and enabling factors (training, consulting guidelines, refresher courses, and knowledge category of the HCWs about MDR-TB-IPC).

### 2.8. Data Quality Management

Two days of training were given to all four data collectors to make them familiar with the tool and research ethical approaches. The first-day session of the training focused on a general overview of any research protocol and its contents, the reviewing and approval process, and ethical and confidentiality issues. On the second day's morning training session, due attention was given to the page-by-page familiarization of the developed questionnaire. On the second day of the final session of the training, a single questionnaire was filled out in the training auditorium room as a team.

Subsequently, a pretest of the questionnaire tool was demonstrated on a small group of HCWs by the researchers, 19 HCWs from different professions and both genders in a nearby health centre to Saint Peter's TB-specialized Hospital, named Addis Hiwot Health Center, to attest the questionnaire user-friendly, free from ambiguity, properly addressing the message designed for whenever administered to the HCWs. Based on the reactions from respondents to pretesting, a few modifications were made. After the research team made the necessary amendments to the pretest feedback, the study settings were communicated in detail to the data collectors. Finally, the corrected questionnaire tool was distributed to the data collectors, where data collector collected 96 completed questionnaires. The collected data were again checked for their completeness by the researchers before the data entry process into the computer software.

### 2.9. Data Analyses

Data were entered into Epi-data version 3.1 and exported to SPSS software. All analyses were performed using SPSS software (Windows version 27, SPSS Inc., Chicago, IL, USA). Descriptive statistics using summaries, frequencies, and percentages were applied to describe the study variables. The descriptive data analysis outputs are presented in the form of tables and graphs. Regarding inferential statistics, bivariate logistic regression analysis was performed to identify candidate variables for multivariable logistic regression model analysis. After the one-on-one variable analysis was carried out between the dependent and independent variables by the bivariate logistic regression models, variables with a *ρ* value less than 0.25 were considered candidates for multivariable logistics regression model analysis. The odds ratio (OR) at a 95% confidence interval (CI) was used to measure the strength of the association between the variables. In the final model, a variable with a *ρ* value of less than 0.05 was considered an independent determinant factor associated with the attitude of HCWs about the IPC of nosocomial MDR-TB in TB-specialized national hospitals of Ethiopia. To identify some other enabling factors independently associated with the attitude of HCWs about the IPC of nosocomial MDR-TB, we estimated the crude odds ratio (COR) and adjusted odds ratio (AOR) at a 95% CI.

### 2.10. Operational Definitions

A healthcare worker (HCW) is a person principally employed to serve TB management and care centres in the two TB-specialized Hospitals, such as a medical doctor, health officer, health education expert, internist, pulmonologist, MLT, radiologist, clinical psychologist, pharmacist, optometrist, nurse, midwifery, clinical social worker, or any other person who is authorized to work/practice by the country's ministry of health and performing within the scope of the hospital authorization within the TB/MDR-TB centre as defined by state law and/or the hospital legislation or regulations.

The mean square is a measurement used to define whether the HCW has a favourable attitude or not.

Attitude is a complex mental status involving beliefs, feelings, and values regarding nosocomial MDR-TB-IPC. It was assessed with nine questions, based on the Likert scale. The scoring was conducted by classifying the responses into two groups. If the right response was agreed and strongly agreed, then a score of 1 is given, otherwise zero. There are five measurement scales for each administered question to assess the attitude of the HCWs. Strongly agree is when for a positive assessment the participant gave 1 point and 0 points for the negative assessment; agree is when for a positive assessment the participants gave 1 point and 0 points for a negative assessment; neutral (neither agree nor disagree) is when for both positive and negative assessments the participants gave 0 points; disagree is when for a positive assessment the participant gave 0 points and 1 point for the negative assessment; and strongly disagree is when for a positive assessment the participant gave 0 points and allotted 1 point for the negative assessment.

The overall attitude is the summary of 9 attitude questions (based on the Likert scale) assessment scores. The mean was considered to classify favourable and unfavourable attitudes.

A favourable attitude is an attitude score equal to or above the mean score (5.78).

An unfavourable attitude is when the mean score is below the mean score (5.78).

Multidrug-resistant TB occurs when MTB is resistant to isoniazid and rifampicin, with or without resistance to other drugs. It means that when strains of MTB are resistant to at least the first-line drugs isoniazid and rifampicin.

Preextensively drug-resistant TB is when strains of MTB are resistant to at least isoniazid and rifampin and either a fluoroquinolone or a second-line injectable agent (but not both) for MTB.

Extensively drug-resistant TB is when the Mycobacterium tuberculosis is resistant to isoniazid and rifampicin, as well as fluoroquinolones (levofloxacin, moxifloxacin, and gatifloxacin) and injectables (kanamycin, capreomycin, amikacin). Or it means that when strains of MTB are resistant to at least isoniazid, rifampin, fluoroquinolones (e.g., moxifloxacin, ofloxacin, levofloxacin, sparfloxacin, gatifloxacin, and ciprofloxacin), and one of the three injectable second-line drugs (amikacin, kanamycin, and capreomycin).

The risk of MTB transmission is the probability of passing MTB to another individual. This may be influenced by factors such as the frequency of contact with the source person, the proximity and duration of contact, the use of respiratory protection, environmental factors (e.g., dilution, ventilation, and other air disinfection), the infectiousness of the source person, and the immune status of the exposed person.

## 3. Results and Discussion

### 3.1. Results

#### 3.1.1. Demographic Characteristics of Study Participants

**Figure 1:**
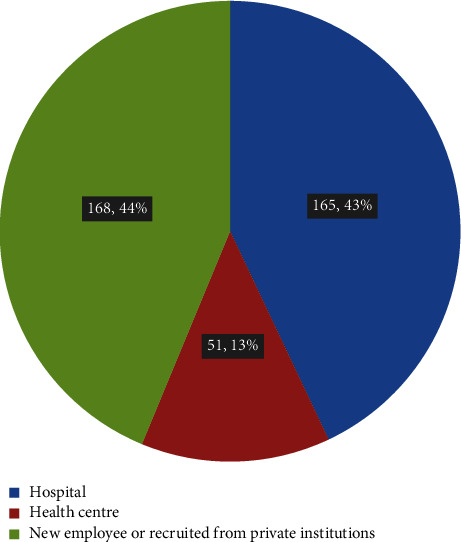
Previous employment facility or status of the HCWs.

Among the 384 participants in this study, the majority of the respondent HCWs, 181 (47.1%), fall between the age range category of 30–40 years, and 208 (54.2%) are male. The majority of them were nurses (153, 39.8%) by profession, followed by 59 (15.4%) medical doctors. More than half of the respondents were single. The majority of the respondent's monthly income (36.7%) was above 11001.00 ETB (on average, one ETB equals 0.023 United States dollars during this study's data collection period). More than half of the study participants (58.6%) had work experience of three or more years (Table 1). Only fifty-one (13.3%) had been working in health centres previously (Figure 1).

#### 3.1.2. TB Diagnosis, Treatment, and Training

Fifty-seven (14.8%) of the respondents were diagnosed with tuberculosis. One hundred forty-nine (81%) of the respondents took refreshment training once a year, and among those who took the training (one hundred eighty-four), one hundred seventy-four (93.5%) thought that the training had an impact on the MDR-TB infection prevention skills (Table 2).

#### 3.1.3. The Attitude of Participants about Nosocomial MDR-TB-IPC

**Figure 2:**
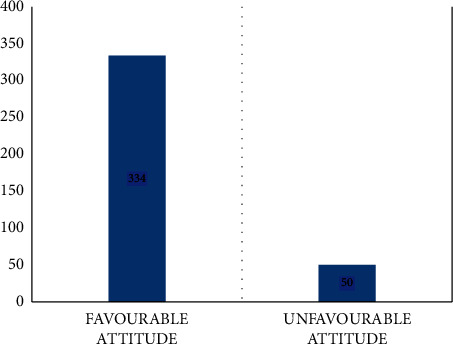
Attitude category of the HCWs regarding MDR-TB-IPC in both hospitals in Ethiopia.

The mean score of the attitude of the 384 study participants was 5.78 (Table 3) with a standard deviation of 1.231. Overall, fifty (13%) of the respondents held unfavourable attitudes (Figure 2).

Two hundred fifty-four (66.1%) of the respondents strongly agreed that most HCWs are afraid that they might contract MDR-TB from the patients. Two hundred (52.1%) of the respondents strongly disagreed that MDR-TB could not be cured, though it could be treated. Attitudinally, two hundred fifty-one (65.4%) of the respondents strongly agreed that cross-ventilation in a room helps to reinforce the IPC approaches against nosocomial MDR-TB. Two hundred twenty-two (57.8%) of the respondents strongly agreed that using N95 or N100 personal protective equipment (PPE) could reduce the risk of MDR-TB transmission (Table 3).

#### 3.1.4. Determinants of HCWs Attitude about Nosocomial MDR-TB-IPC

In the analyses of attitude-related factors, sex, experience, and having good knowledge of infection control for MDR-TB failed to be significant. While, monthly income and previous training received remained significantly associated with favourable attitude scores. Those who earned a monthly income between 7001 and 9000 had a greater (3 folds increase) likelihood of having a favourable attitude towards prevention and control of nosocomial MDR-TB than those who had collected a monthly income of less than 5000 ETB with an AOR of 3.34 at 95% CI (1.11, 10.05) (Table 4). The likelihood of a favourable attitude among the HCWs was as high as 3 times higher in HCWs who received any prior training related to MDR-TB-IPC, with an AOR of 2.96 at 95% CI (1.32, 6.62). As a result, it is clearly shown that those who have prior training, reasonably get a moderately higher monthly income in the form of salary, and have good knowledge scores (Table 4), have a favourable attitude towards MDR-TB infection control and prevention.

### 3.2. Discussion

Multidrug-resistant tuberculosis is an increasing threat to humanity, especially in countries with a high burden of HIV/AIDS. Nosocomial infection control strategies could prevent nearly half of XDR-TB cases, even in a resource-limited setting. However, drug-resistant TB transmission continues spreading, indicating the need to develop and implement control and prevention mechanisms in all settings by increasing awareness through training of all stakeholders and community education [27]. Mycobacterium tuberculosis transmission risk is immense in healthcare settings, particularly TB/MDR-TB treatment and rehabilitation facilities. Nevertheless, the magnitude of the risk varies between settings, the healthcare occupation task force, the prevalence of TB in the community, the patient population, and the knowledge and implementation of MDR-TB-IPC measures. Healthcare-associated transmission of MTB is a direct function of close contact with diseased persons during aerosol-producing procedures such as bronchoscopy, endotracheal intubation, suctioning, open abscess irrigation, autopsy, sputum induction, and aerosol treatments that induce coughing [28].

Overall, in this study, 87% of the study respondents had a favourable attitude towards the nosocomial MDR-TB-IPC. Previous exposure to refreshment training and collecting a monthly income range of 7001–9000 ETB in the form of salary were found to be independent predictors (statistically significant) of favourable attitudes about IPC infection prevention and control among the HCWs operating in the MDR-TB centres of the two nationally well-knownTB-specialized public hospitals of Ethiopia. The trend of knowledge score among HCWs in this study was in harmony with that of attitude score but not significantly associated with it.

The current report's findings were somehow higher than the previous result reported from Addis Ababa long ago, where the authors reported that the HCWs felt that they were at high risk for occupational acquisition of TB (71%) [24]. The difference between the two studies might be due to the differences between the study settings, TB-specialized hospitals in the current study versus general purpose teaching hospitals in the later study. Also, the present study with the overall favourable attitude score of HCWs (87%) is in line with the previous study from Boru Meda hospital in the Amhara regional state of Ethiopia [29], where the majority of the respondents (76.6%) had a favourable attitude, and with a study conducted on the attitude of HCWs regarding MDR-TB infection control and prevention in the Delta State of Nigeria (71.9%) [30]. Though different, comparable result (73.2%) was reported from Nepal [28] and Mecca, Saudi Arabia, among healthcare workers during the 2016 Hajj (with a mean attitude score of 73%) about the attitude of HCWs towards nosocomial TB-IPC [31].

In contrast, a previous study on healthcare providers' attitudes towards TB in a pastoralist community in Ethiopia reported only about 44.4% of HCWs had favourable attitudes toward TB control and prevention [32]. In contrast to our study, a marginally high unfavourable attitude score (76.5%) was reported from a study conducted on attitudes toward drug-resistant TB in the Eastern Cape Province of South Africa [33], though the target population group of the study was the general community members. The similarity between the present study and the previous studies might be due to the similarity of the target population. On the other hand, the discrepancies between the current study and the previous studies might be because of the differences in terms of the measurements, sample size, study design, and study settings. Besides, for the differences, as an example, the South African Port Elizabeth was conducted in a limited area, and the sample size was not chosen by random sampling methods (rather convenience sampling method). On top of these, the frequency of exposure to the latest information, training, and national MDR-TB policies among the current study participants might upscale the proportion of the attitude score in the two national TB-specialized public hospitals, Saint Peter's and ALERT TB-specialized hospitals in Ethiopia.

A comparable attitude to our study, a previous study on attitudes regarding tuberculosis amongst healthcare workers in Moyen-Ogooué Province of Gabon revealed that attitudes were generally positive toward tuberculosis infection control efforts. Healthcare workers reported that infection control measures were not consistently practiced; 72.8% (75/103) of the participants were scared of becoming infected with tuberculosis; and 98.1% saw a need for improvement of local tuberculosis control [34]. Notable sociopolitical and policy influences impacting TB-IPC implementation include stigma against TB and the availability of facility-specific TB-IPC policies, respectively. Hardware influences on TB-IPC implementation referred to availability, knowledge, and educational development of staff, timeliness of service delivery, availability of equipment, such as respirators and masks, space for patient separation, funding, and TB-IPC information, education, and communication materials and tools. Commonly reported health system software influences were workplace values and established practices, staff agency, TB risk perceptions, and fears as well as staff attitudes towards TB-IPC [35]. Theoretically, the nosocomial transmission of TB from index person to HCWs is estimated or the probability (**P**) of transmission of tuberculosis to health care workers can be approximated by the equation *P*=1 − *e*^−**I***qpt*/*Q*^, where **e** = denotes exponential, **I** = the number of patients with active tuberculosis in contact with a worker, **q** **=** the infectiousness of the index case, ***p*** **=** the ventilation rate of the worker, ***t*** **=** the duration of exposure, and **Q** = the air-exchange rate in the interior space [36]. The chain of bacilli transmission from patients to HCWs is established by DNA fingerprinting of isolates of bacilli showing similar DNA patterns from both the patient and the exposed HCWs [37].

In this study, the attitude score of the HCWs towards nosocomial MDR-TB-IPC was not significantly influenced by most of the sociodemographic characteristics measured, but rather by the monthly income they earned. However, a poor association between income and PTB was reported from a study in Taiwan, as there was not much variation in odds ratios across the income quintiles evaluated [38]. Another study on the attitude of HCWs versus socioeconomic variables in Georgia showed that only 52% and 48% of the HCWs were willing to undergo annual TB infection screening and TB infection treatment, respectively. In multivariate analysis, HCWs who worried about acquiring MDR-TB infection (AOR = 1.7; 95% CI, 1.28–2.25), who thought screening contacts of TB cases is important (AOR = 3.4; 95% CI, 1.35–8.65), and who were physicians (AOR = 1.7; 95% CI, 1.08–2.60), were more likely to accept annual TB infection screening. With regard to TB infection treatment, HCWs who worked in an outpatient TB facility (AOR = 0.3; 95% CI, 0.11–0.58) or perceived a high personal risk of TB reinfection (AOR = 0.5; 95% CI, 0.37–0.64) were less likely to accept TB infection treatment [39].

In the present study, the attitude score of the HCWs towards nosocomial MDR-TB-IPC was significantly influenced by the refreshment training they gained (AOR = 2.96; 95% CI: 1.32–6.62). Other studies also support the current study findings, such as [40] in Uganda, which reported that having not attended TB-IPC training was significantly associated with low self-efficacy (AOR = 0.52; 95% CI: 0.33–0.81) and a low perceived threat of acquiring TB infection at work (AOR = 0.54; 95% CI: 0.36–0.81), and [41] in Southwest Nigeria, which showed that the use of trained community volunteers to share information on TB improved the overall attitudes of the respondents. The similarity between the current study and those studies elsewhere might be because of the commitment of the WHO to high TB/MDR-TB burden countries in Africa.

On the other hand, our study findings concerning the attitudes of HCWs with that of the prior on-job training are in contrast with those reported in previous studies, such as [42] in Odi district hospital in South Africa, which affirmed that training was not associated with doctors' attitudes towards TB-IPC [43], in Mizan Tepi University Teaching Hospital in Ethiopia, which noted that the history of TB infection prevention and control training was not a significant predictor of the attitude of the HCWs [31], in Mecca, Saudi Arabia, during the 2016 Hajj, which reported that previous training was not associated with attitude scores of HCWs but with age and occupation [44], and in the Kingdom of Saudi Arabia revealed results stating that there were no significant differences in the attitudes of the participants (primary healthcare professionals) with their sociodemographic characteristic and those who got a prior training program about infection control. The discrepancy is primarily due to sample size and target population differences.

Generally, the effect of training could be explained by the fact that training related to MDR-TB increases or refreshes the HCWs attitude and knowledge, which subsequently clarifies vague perceptions regarding MDR-TB-IPC. Thus, the resultant training outcomes and impacts are to erode the uncertainty about MDR-TB and bring about a favourable attitude among HCWs. An added positive attribute of the HCWs attitude in the current study was their participation in MDR-TB-related patient education and community awareness campaigns in an attempt to cease the transmission cycle of infection.

This study has limitations, such as that practice competency was approximated by self-reporting, it was conducted in hospitals assumed to be ideal in terms of TB/MDR-TB-IPC, administrative staff were excluded, an assessment of the incidence of latent TB in HCWs has not been conducted, training of HCWs after the research conclusion was not executed, a practical assessment of existing IPC measures in the hospitals was not implemented, and gender balancing was not made during the data collectors' selection. Nonetheless, this study offers novel findings that may be applied to similar settings in LMICs. To mention a few of those strengths, the large sample size, thorough analyses, and representativeness in all regards were among others.

## 4. Conclusions

In the present study, the mean score of the attitude among the 384 healthcare workers (HCWs) is 5.78 with a standard deviation of 1.231. Overall, fifty (13%) of the HCWs held unfavourable attitudes about nosocomial multidrug-resistant tuberculosis (MDR-TB) infection prevention and control (IPC). Our study finds moderate attitudes among HCWs towards nosocomial MDR-TB-IPC. While the results of the study are encouraging at primary healthcare institutions levels, important attitude gaps are visible in the two TB-specialized public hospitals in Ethiopia. Gaudily, the overall attitudes of HCWs on nosocomial MDR-TB-IPC are not satisfactory at the two top-rankedTB-specialized public hospitals, as these hospitals are mandated to develop and/or amend the country's TB/MDR-TB strategies, policies, and guidelines. This calls for multifaceted interventions to improve the attitude score of HCWs regarding nosocomial MDR-TB-IPC, including tailored, periodic TB education and training aimed at boosting favourable attitudes and improving HCWs behaviour. Hence, effective and stringent IPC measures, including regular skill-based training and orientation for all categories of HCWs, can improve infection control attitudes in health facilities. Particularly, the training of HCWs on IPC should emphasize the nonclinical staff due to their limited grasp of IPC measures. Additionally, addressing HCWs social needs, such as a reasonable increment in their monthly salary, through the political will of multisectorial institutions' actions. Moreover, implementing uninterrupted monitoring and evaluation of the attitudes of the workforce managing MDR-TB in health facilities should be in place to lead IPC efficiently at all public hospitals.

## Figures and Tables

**Table 1:** Demographic characteristics of the study participants.

Variables	Categories	Count	Percent
Age (in years)	20–30 years	108	28.1
	30–40 years	181	47.1
	41 and above years	95	24.7

Sex	Male	208	54.2
	Female	176	45.8

Marital status	Single	231	60.2
	Ever married	153	39.8

Profession	Medical doctor	59	15.4
	Nurse	164	42.7
	Pharmacy expert	28	7.3
	Laboratory technologist	35	9.1
	Radiologist	14	3.6
	Midwives	15	3.9
	Health officer	15	3.9
	Janitor	29	7.6
	Others^©^	25	6.5

Monthly income (in ETB)	5000 and less	81	21.1
	5001–7000	39	10.2
	7001–9000	68	17.7
	9001–11000	55	14.3
	11001 and above	141	36.7

Work experience in the current hospital	<3 years	159	41.4
	≥3 years	225	58.6

Under profession, Others^©^ **=** clinical psychologists, optometrists, orthopedists, dentists, EENT specialists, dermatologists, runners/porters, epidemiologists, health education experts, and nutritionists.

**Table 2:** Background general information responses of the HCWs to complement their attitude.

Characteristics	Responses	Count	Percent
Ever been diagnosed with TB	Yes	57	14.8
	No	327	85.2

Ever been treated with anti-TB drugs	Yes	57	14.8
	No	327	85.2

Ever been participated in a training program about MDR-TB	Yes	184	47.9
	No	200	52.1

Frequency of training among those received	Once a year	149	81
	Twice a year	4	2.2
	Thrice a year	13	7.1
	Four-time or more	18	9.8

Training has an impact on MDR-TB infection prevention skills	Yes	174	93.5
	No	10	6.5

Knowledge score	Good (measured)	346	90.1
	Poor (measured)	38	9.9

**Table 3:** Attitude of HCWs about nosocomial MDR-TB-IPC in the two hospitals in Ethiopia.

Attitude-related measurements	Categories	Count	Percent
Most HCWs are afraid of contracting MDR-TB from patients	Strongly agree	254	66.1
	Agree	85	22.1
	Neutral	33	8.6
	Disagree	9	2.3
	Strongly disagree	3	0.8

MDR-TB patients cause the problem by themselves	Strongly agree	84	21.9
	Agree	94	24.5
	Neutral	63	16.4
	Disagree	108	28.1
	Strongly disagree	35	9.1

Social and cultural factors constitute treatment barriers	Strongly agree	199	51.8
	Agree	141	36.7
	Neutral	30	7.8
	Disagree	12	3.1
	Strongly disagree	2	0.5

Traditional or alternative medicine worsen the treatment of MDR-TB	Strongly agree	150	39.1
	Agree	96	25
	Neutral	72	18.8
	Disagree	23	6
	Strongly disagree	43	11.2

MDR-TB cannot be cured through treatment	Strongly agree	24	6.2
	Agree	21	5.5
	Neutral	38	9.9
	Disagree	101	26.3
	Strongly disagree	200	52.1

MDR-TB patients should be allowed to die without treatment	Strongly agree	0	0
	Agree	0	0
	Neutral	23	6
	Disagree	92	24
	Strongly disagree	269	70.1

Cross-ventilation in a room helps in infection control attitude	Strongly agree	251	65.4
	Agree	108	28.1
	Neutral	12	3.1
	Disagree	7	1.8
	Strongly disagree	6	1.6

Patients' education and increasing community awareness about MDR-TB helps in the control of the disease	Strongly agree	305	79.4
	Agree	70	18.2
	Neutral	3	0.8
	Disagree	3	0.8
	Strongly disagree	3	0.8

Using N95 or N100 could reduce the risk of MDR-TB transmission	Strongly agree	222	57.8
	Agree	114	29.7
	Neutral	41	10.7
	Disagree	1	0.3
	Strongly disagree	6	1.6
	Total	384	100

The overall mean score of HCWs attitude	5.78 and above	334	87
	Below 5.78	50	13

**Table 4:** Attitude predictor factors among HCWs about the nosocomial MDR-TB-IPC.

Variables	Category	Attitude (%)		*P* value	COR (95% CI)	*P* value	AOR (95% CI)
		Good	Poor				
Gender	Male	189 (90.9)	19 (9.1)	0.001		0.249	1
	Female	145 (82.4)	31 (17.6)	0.15	0.470 (0.25–0.86)		0.67 (0.35–1.31)

Monthly income	<5001	58 (71.6)	23 (28.4)	0.001	1	0.306	1
	5001–7000	34 (87.2)	5 (12.8)	0.06	2.69 (0.94–7.75)	0.290	1.84 (0.596–5.66)
	7001–9000	63 (92.6)	5 (7.4)	0.002	**4.99 (1.78–14.01)**	0.032^*∗*^	**3.34 (1.11-10.05) ** ^*∗*^
	9001–11000	49 (89.1)	6 (10.9)	0.018	3.24 (1.22–8.59)	0.388	1.60 (0.55–4.66)
	>11000	130 (92.2)	11 (7.8)	0.001	4.69 (2.14–10.25)	0.297	1.65 (0.64–4.27)

Experience	<3 years	222 (84.4)	41 (15.6)				
	≥3 years	112 (92.6)	9 (7.4)	0.031	**2.29 (1.08–4.89)**	0.249	1.63 (0.71–3.74)

Training received	Yes	174 (94.6)	10 (5.4)	0.001	**4.35 (2.11–8.98)**	**0.008 ** ^*∗*^	**2.96 (1.32-6.62) ** ^*∗*^
	No	160 (80.0)	40 (20.0)	0.001	4		1

Knowledge level	Poor	24 (63.2)	14 (36.8)	0.001		0.056	1
	Good	310 (89.6)	36 (10.4)		**5.02 (2.38–10.57)**		2.35 (0.98–5.64)

^*∗*^ *P* value < 0.05; COR, crude odds ratio; AOR, adjusted odds ratio.

## Data Availability

The datasets used and/or analyzed during the current study are available from the corresponding author upon request.

## Abbreviations

AIDS: Acquired immunodeficiency syndrome
ALERT: All African Leprosy Research and Training
DOTS: Directly observed treatment short course
HCWs: Healthcare workers
HIV: Human immunodeficiency virus
IPC: Infection prevention and control
IRB: Institutional review board
LMICs: Low- and middle-income countries
MTB: *Mycobacterium tuberculosis*
MLT: Medical laboratory technologist
MDR-TB: Multidrug-resistant tuberculosis
OPD: Outpatient department
RR/MDR-TB: Rifampicin-resistant or multidrug-resistant tuberculosis
SDG: Sustainable development goal
SPSS: Statistical products for social science
TIC/TFC: Treatment initiating center/treatment follow-up centre
TB: Tuberculosis
WHO: World Health Organization
XDR-TB: Extensively drug-resistant tuberculosis.
